# Corona pan(dem)ic: gateway to global surveillance

**DOI:** 10.1007/s10676-020-09569-5

**Published:** 2020-10-20

**Authors:** Regina Sibylle Surber

**Affiliations:** 1 Center for Ethics, University of Zurich, Zollikerstrasse 117, 8008 Zurich, Switzerland; 2 ICT4Peace Foundation, Pfalzgasse 1, Zurich, Switzerland

**Keywords:** Corona, Covid-19, Pandemic, Human rights, Digital technologies, Surveillance, Ethics

## Abstract

The essay reviews the digital emergency measures many governments have adopted in an attempt to curb Covid-19. It argues that those ‘virologically legitimized’ measures may infringe the human right to privacy and mark the transition into a world of global surveillance. At this possible turning point in human history, panic and latent fear seem to fog much needed farsightedness. Leaving the current state of emotional paralysis and restarting to critically assess the digital pandemic management can serve as an emergency break against drifting into a new era of digital monitoring.

It is said that the ‘corona crisis’ may be the biggest crisis of the current generation. As of 28 September 2020, 32.7 million persons are said to have been tested positive on Sars-CoV-2 in more than 200 countries and territories, and 991.000 people are said to have died from Covid-19 (WHO 2020a). On 11 March 2020, the World Health Organization’s Director General declared Covid-19 as a pandemic (WHO 2020b). By the end of January and early February 2020, a wave of panic of the previously unknown physical Covid-19 illness has spread across the planet.^1^

## Governmental restrictions and human rights

In an attempt to contain the spread of the corona pandemic, and in order for national health care systems not to be overwhelmed by the potentially enormous influx of people suffering from the acute respiratory syndrome that Sars-CoV-2 may trigger, many governments have adopted emergency measures to secure public health and order.

Those emergency measures are, arguably, drastic. As of March 2020, almost the entire globe ‘locked down’:

Most governments have temporarily closed educational institutions, impacting 60% of the world’s student population. Several other countries have implemented localized closures that may impact millions of additional learners (UNESCO 2020).^2^

As a result of the pandemic, around 70 countries across the world had imposed or still are imposing entry bans, quarantines and other restrictions for citizens or travelers to most affected areas (Salcedo and Cherelus 2020). As of 28 September 2020, around 70 countries and territories still impose global restrictions applying to all foreign countries, or prevented their citizens from travelling (IATA 2020). Many governments had also implemented curfews or urged people to stay at and work from home. In places where people were still allowed to leave their houses, gatherings of more than a handful of people were banned.^3^ During the lockdowns, in many countries, doctors’ offices and pharmacies remained open, but restaurants, bars and non-essential shops in the majority of places around the globe were ordered to close their doors. This threatened the existence of small companies, with some businesses already declaring insolvency as early as March 2020 (Allen 2020), many people becoming unemployed,^4^ and governments adopting economic support measures of unprecedented amounts (European Commission 2020). Further, especially in low-income countries, health access was restricted to almost Covid-19-only cases, disrupting the prevention and treatment of other noncommunicable diseases (WHO 2020c).^5^

Whereas lockdowns are gradually eased and terminated in phases,^6^ fears of a second virus wave are currently spreading again due to surges in the number of confirmed cases in various regions. This pushes some countries to consider a retake on restrictions^7^ or even mandating second lockdowns.^8^

The restrictions affect our human rights. Curfews and the ban on gatherings may infringe our freedoms of movement^9^ and assembly.^10^ The closing of educational institutions worldwide severely rephrases access to education, a right granted by the Universal Declaration of Human Rights (UDHR).^11^ What is more, the requirement to shift to online-learning exposes education’s digital divide: in poorer countries, children may not have the resources required to be digitally home-schooled (Thong 2020). Further, many health institutions and hospitals have been forced to triage patients in case of sudden overloads, which may impede the right to access to medical care.^12^ In addition, the shutting down of public life has put jobs and livelihoods into severe jeopardy—possibly affecting our right to work.^13^

What is more, the listed emergency measures have forced a great majority of people to physically isolate and distance from loved ones. Millions of people also face economic turmoil, because they have lost, or are at risk of losing, income and livelihood.^14^ Misinformation and general unknowingness about the virus create deep uncertainty about the future. This may probably entail a long-term upsurge in the severity and the number of mental health problems (UN 2020). Furthermore, first research findings indicate that many children suffer from severe psychological stress and the loss of education due to the closing of schools.^15^ Moreover, with unemployed people, suicide rates are generally higher, and the quality of life lower (Kroll and Lampert 2012). Hence, in attempting to secure public *physical* health, governmental restrictions may well be read as potentially putting public *mental* health into jeopardy.

## Global surveillance

Besides restrictions on physical movement that entail the above-mentioned potential risks to our freedoms of movement and assembly, our rights to access education and health institutions, and our right to work, many governments also rely on emerging technologies in their ‘fight’^16^ against the pandemic. Those ‘digital measures’ may severely infringe our human right to privacy,^17^ and may mark the transition into a world of surveillance technology.

The adopted emergency measures that engage new technologies aim primarily at analyzing the spreading pattern of the virus and at monitoring and enforcing curfews. Through relying on digital strategies, governments follow the World Health Organization’s recommendation to trace contacts between their citizens (WHO 2020d).

The emergency measures engaging new technologies may be roughly divided into five groups^18^:

### Contact tracing apps

Contact tracing apps are designed to support curbing the spread of Sars-CoV-2 by tracking individuals and those they have come into contact with. Usually, if a person was found to be infected, the people she has been recently in contact with are informed. Often, they are then asked to self-quarantine. As of 3 July 2020, roughly 50 countries have been using contact tracing apps in dealing with corona: Australia, Austria, Azerbaijan, Bahrain, Bangladesh, Brunei, Bulgaria, Canada (Alberta), China (Tangermann 2020), Cyprus, Czech Republic, France, Georgia, Germany, Ghana, Hungary, Iceland, India, Indonesia, Iran, Israel, Italy, Japan, Jordan, Kyrgyzstan, Latvia, Malaysia, Mexico, New Zealand, North Macedonia, Norway, Peru, Philippines, Poland, Qatar, Saudi Arabia, Singapore, Slovakia, South Africa, South Korea, Spain, Switzerland, Thailand, Tunisia, United States of America, United Arab Emirates, Ukraine, United Kingdom, Uruguay, and Vietnam.^19^ According to research conducted by TopVPN.com, about one third of the apps rely on GPS technology, a third on Bluetooth, and another third use both Bluetooth and GPS (Woodhams 2020).

### Digital tracking

Digital tracking includes the use of aggregated mobile location data to track citizens during lockdowns, apps designed to help identify the location of those with Sars-CoV-2,^20^ and the deployment of advanced mobile monitoring technologies. As of 3 July 2020, 31 countries around the world have adopted digital tracking measures. E.g. government officials across the US are relying on location data from millions of cellphone users to better understand the movements of Americans during the pandemic, and how those movements may be affecting the disease (Tau 2020). The British government is working with major mobile network O2 to analyze its users’ location data (Martin 2020). Other countries whose governments retrieve or had retrieved their citizens’ geolocation data are Argentina (Davidovsky 2020), Austria (Mijnssen 2020), Belgium (Cloot 2020), Brazil (Mari 2020), Bulgaria,^21^ China (Davidson 2020), Ecuador (EcuadorTV 2020), Finland (Telia 2020), Germany (Reikowski 2020), Guatemala (Estrada Tobar 2020) Hong Kong (Hui 2020), India (Srivastava and Nagaraj 2020), Iran (Gilbert 2020), Israel (Reuters 2020a), Italy (Vodafone 2020), Jordan,^22^ Kazakhstan (Gussarova 2020), Morocco (Chahir 2020), New Zealand (Andelane 2020), Pakistan (Jahangir 2020), Poland (Privacy International 2020), Russia,^23^ Singapore (Baharudin 2020), South Africa (Business Insider SA 2020), South Korea (Kim 2020), Spain (GovLab 2020), Switzerland (Reuters 2020b), Taiwan (Chen 2020), and Turkey (HRW 2020a).

### Physical surveillance

In order to slow the spread of Covid-19, governments are also adopting increasingly extensive physical surveillance measures. Those measures include the deployment of facial recognition cameras equipped with heat sensors, surveillance drones used to monitor citizens’ movements, and extensive CCTV (Closed Circuit Television) networks. As of 3 July 2020, 11 countries have been using physical surveillance technologies to address Covid-19. The West Australian police force (Spires 2020), the New York Police Department,^24^ UK police forces,^25^ Belgian police,^26^ and Madrid’s police force^27^ are increasingly relying on the use of aerial footage through drones in order to enforce ongoing lockdowns and monitor citizen movements. Since the corona virus outbreak, also Russia (Reuters 2020c) and China (Kuo 2020; Shen 2020) are relying on a host of extensive surveillance mechanisms, including both drones and facial recognition cameras. Other countries using physical surveillance are the Bahrain (McArthur 2020), France (BBC), India,^28^ and the United Arab Emirates (Al Monitor 2020).

### Censorship

Since the outbreak of the corona virus, there has been an acceleration in the spread of false information (Woodhams 2020). In order to control and contain mis- and disinformation, governments have sought to regulate online content and promote official facts and figures from international health organizations. However, as of 3 July 2020, 18 governments have used the rise of mis- and dis-information about Covid-19 to justify censorship practices that aim at silencing regime critics and at controlling the flow of information. E.g. Cambodian (HRW 2020b) and Ugandan (Unwanted Witness 2020) authorities have arrested social media platform users that spread info about the virus. In Niger (CPJ 2020), authorities have arrested a journalist due to his coverage of the virus. Egypt (Al Jazeera 2020a) has taken away the press credentials of a British Journalist due to his alleged bad faith in how Egypt is dealing with the virus. Iran (Paganini 2020) blocked access to the Farsi language edition of Wikipedia due to criticism on how its authorities are handling the pandemic. Further countries leveraging the risk of false information about corona for censorship purposes are Azerbaijan (RSF 2020c), Bangladesh (RSF 2020a), China (Ruan et al. 2020), Hong Kong,^29^ Japan (Denyer 2020), Kenya (Woodhams 2020), Russia (RFE 2020), Singapore (Mahtani 2020), Thailand (HRW 2020c), Turkey (RSF 2020b), Turkmenistan (RSF 2020e), Venezuela (Cincurova 2020), and Zimbabwe (RSF 2020d).

### Internet shutdowns

During the spread of a novel virus, access to and a free flow of reliable and correct information is urgent. Still, the governments of Bangladesh (HRW 2019),^30^ Ethiopia (AFP 2020a), India (Ganai 2020), and Myanmar (Al Jazeera 2020b) have restricted internet access some areas of their territories.

The description of ongoing monitoring and surveillance measures leads to two observations. First, our right to privacy may be severely infringed. And second, for the first time in human history, technology may make it possible to monitor almost everybody, almost everywhere, almost all the time. In other words, the corona panic and pandemic may let us slide into a world of global surveillance. Most unfortunately, due to the level of fear and panic, we seem to accept or even take part in those measures without the usual reflex of questioning them.

## Potential permanence and inefficacy of emergency surveillance measures

In an exceptional situation, states may need additional powers to secure public safety and health. National constitutions as well as international human rights treaties^31^ contain clauses that allow governments to temporarily suspend some of their obligations during a time of crisis. In those situations, governments can invoke special powers that would normally be considered infringements on human rights, even without formally declaring a state of emergency.^32^ However, those powers are not absolute. Emergency measures must be *legal*^33^ and *proportionate*,^34^ as well as *necessary* and *time-bound*. What is more, government authorities carry the burden of justifying the restrictions (OHCHR 2020).

Restrictions must be *necessary* for the protection of public health. Most importantly, emergency measures can qualify as necessary only if they are also *efficacious*. An instrument is efficacious if it produces the intended effect. An instrument that is incapable of producing the intended effect, is, hence, not efficacious and cannot be necessary for achieving that effect. It follows that, in order to determine whether surveillance mechanisms can qualify as necessary measures, one must determine whether those measures can actually provide *reliable* and *useful* location information, i.e. whether they are efficacious.

Especially measures tapping personal smartphone information could not prove fully efficacious. How can cell phones be tracked? Cell phone towers are one option, but they provide only a very rough measure that is not useful to determine whether, e.g. a six-foot-proximity threshold is abided by. GPS signals are finer, but they work only outside, and can, therefore, not determine whether two people, e.g. sat in the same train wagon. What is more, as GPS drains battery, many people have it turned off in the first place. A WIFI network or Bluetooth beacon to which a smartphone is connected is a further location indicator. Still, the fact that two cell phones are connected to the same WIFI or Bluetooth does not say that they are not keeping a six-foot distance (Landau 2020; Stanley et al. 2020). Given that the majority of contact tracing apps rely on Bluetooth and GPS, those observations raise the question of those apps’ effectiveness and, hence, necessity.

Besides the requirements of legality, proportionality, necessity, and non-discrimination, emergency legislation must be *time-bound.*^35^ Unfortunately, crises have a habit to fast-forward certain processes and instruments, whose consequences may not disappear once the crisis is over. Hence, the surveillance measures endangering, in particular, our human right to privacy may not be terminated once the pandemic is successfully contained. Although lockdowns are being terminated now, the above-listed apps and digital instruments are, largely, still in place. Hence, the requirement of time limitation may well be neglected.

Two considerations may support the danger of persisting digital surveillance: On the one hand, digital surveillance could create financial pay-offs. If anything in the world is growing exponentially today, it is the provision of and the access to personal data. This may fuel the AI industry and could partially support the economic recovery once the virus spread is curbed.^36^ What is more, the ‘digital pandemic shock doctrine’ does not only cover virus containment strategies or the monitoring and enforcing of curfews. Forced social distancing and isolation, and the shutting down of every-day institutions such as schools and workplaces, are a breeding ground for technologies that aim at re-installing our entire social life in the digital space. Our months-long pandemic isolation may well be a lab for a permanent contactless future of telehealth, broadband, and remote learning—highly profitable for businesses developing those services.

On the other hand, surveillance technologies may persist if people spread the perspective of the next crisis being ‘just around the corner’. The speed of the Covid-19 panic wave was enormous, and the paralysis of reflection it created severe. Pre-emptive fear may corroborate and consolidate national and global surveillance mechanisms, and may make us blind to our duty to question them.

## Panic and legitimation

The thought driving the rather precipitous governmental behavior can be summarized as follows: ‘Any measure necessary to save *humanity* is legitimate.’^37^ This clause begs the question what the term ‘humanity’ means in this very context:

On the one hand side, ‘humanity’ can refer to the ‘human species’. In this case, the pressing question is: Could Sars-CoV-2 extinguish the human species? First estimates of Spring 2020 declared a Covid-19 lethality of approximately 10% (EBM 2020). Meanwhile, those figures have been proved wrong. Today, experts estimate a so-called ‘Infection fatality rate’^38^ of between 0.27 and 0.36% (Ioannidis 2020; Streeck et al. 2020).^39^ This estimate is considerably lower and draws a much less fatalistic picture of the pandemic. However, it must be noted that, as of today, there still exists only very little robust medical evidence on Covid-19, and further studies are necessary (EBM 2020).

In order to understand the spreading pattern of the virus, many countries are currently also conducting population-wide-testing.^40^ The most common way of testing for Covid-19 is the so-called PCR-test. In some countries both people who do show as well as people who do *not* show clinical signs of SARS-CoV-19 are tested.^41^ This sort of indiscriminate testing, however, may lead to excessive diagnostic. Furthermore, as of today, there exist no published and medically usable studies on the accuracy of the PCR-test.^42^ Moreover, and most interestingly, whereas the numbers of persons tested positive are currently on the rise in certain countries, those states’ number of hospitalized patients requiring intensive care remains relatively static (EBM 2020). Hence, not only is there only little robust medical evidence on Covid-19, but the informative value of the test results may not be fully reliable.

Those observations allow the assumption that ‘humanity’, understood as the human species, may, most probably, not be at risk of extinction by Covid-19. The clause ‘any measure necessary to save humanity’ may, hence, not legitimize the rush into both digital and non-digital governmental emergency measures against the current pandemic.

On the other hand, ‘humanity’ may also refer to what may slumber within each individual person. The protection of this very seed of humanity is the task of human rights; and the protection of human rights is the primary task of states. Hence, if ‘humanity’ is understood in this way, the justification for governmental emergency measures may be even more fragile. For, as has been shown, many of those measures, amongst them increased surveillance, may violate certain fundamental human rights. Thus, if ‘humanity’ is understood as each individual human’s value and potential, one could say that it *is*, currently, at risk—not primarily by Covid-19, but by certain governmental emergency measures.

Though it seems improbable that Covid-19 may extinguish the human race, the initial global panic does not seem to fade, but rather to be replaced by a latent and passive fearfulness. One important reason may be that the current risk communication is not based on the medical evidence that would draw a less fatalistic picture. On the one hand side, governmental measures themselves may instill and aggravate fear. As, for many citizens of the globe, the extent and the severity of those interventions are largely unprecedented and, thus, themselves a great shock. On the other hand, also media coverage seems to corroborate the global state of fear. In German-speaking countries, e.g. public reporting does not consistently differentiate between people tested positive on Covid-19, and those who have fallen ill. This differentiation is especially crucial in countries where the rising number of people who are tested positive is not accompanied by a parallel increase in hospitalizations and intensive care treatments.^43^ Furthermore, SARS-CoV-2 incidents are very often reported as absolute numbers without reference figures, and the total numbers are published cumulatively. This contradicts the basic principles for presenting epidemiological data (EBM 2020). This sort of pandemic coverage can be strongly misleading and fuel both individual and societal fear.

If one accepts a civilian duty to reflect upon whether government measures could infringe human rights, the global state of excessive emotionality, or the gradual slide into a latent passivity, must again be replaced by a state of reason—especially given emergency surveillance’s potential permanence. Put differently, not only the pandemic, but also the panic must stop. If not, humans will be incapable to reasonably reflect on whether, and if so, how, to opt out of the path fear has been pushing them on to. What is more, the emerging picture of wide-spread mental health issues due to the demanded isolation and panic may now require an even stronger effort to reclaim both people’s individual willingness as well as their capacity for clear-sightedness.

The responsibility lies with every individual. Any institution is only as strong as the reflected minds of its members and the reflected minds of the population it aims to represent. This conclusion must, first and foremost, guide media professionals. Their responsibility to curb fear and provide well-balanced facts in order to push global society back to reason is enormous. Second, any governmental anti-pandemic decision must be appropriately accompanied by science and, wherever already possible, scientifically substantiated. Only then is it possible to properly document the ratio between the benefit that those measures bring to public health, and the societal costs they entail. Finally, this conclusion must guide anyone whose primary needs are currently still met. If humanity wants to move into a balanced future, humans must both reconquer and *use* their individual reflective capacity, which requires time and quietness. This may have been one profound advantage of the demand to stay at home, as both were, or still are, more easily accessible. We must regard our more isolated and socially cautious lives as an invitation for introspection, for an increased level of self-understanding, and for a new prioritization of values.

## Data Availability

Provided in the essay.

## Appendix: List of Contact Tracing Apps per Country

**Table:**

Australia	*COVIDSafe*
Austria	*Stopp Corona*
Azerbaijan	*e-Tabib*
Bahrain	*BeAware Bahrain*
Bangladesh	*Corona Tracer BD*
Brunei	*BruHealth*
Bulgaria	*ViruSafe*
Canada (Alberta)	*ABTrace Together*
China	*Close Contact Detector*
Cyprus	*CovTracer*
Czech Republic	*Mapy.cz, eRouska*
France	*StopCovid France*
Georgia	*Stop Covid*
Germany	*Corona-Warn App*
Ghana	*GH Covid-19 Tracker*
Hungary	*VirusRadar*
Iceland	*Rakning C-19*
India	*Aarogya Setu, SAIYAM—Track & Trace Together, COVID CARE, Covid Locator, Corona Watch, MahaKavach, COVID-19 Odisha, SMC COVID-19 Tracker, COVID-19 Quarantine Monitor Tamil Nadu, UP Self-Quarentine App, Uttarakhand CV 19 Tracking System*
Indonesia	*PeduliLindungi*
Iran	*Mask.ir*
Israel	*Track Virus, ‘The Shield’*
Italy	*Immuni*
Japan	*COCOA—COVID-19 Contact App*
Jordan	*AMAN App*
Kyrgyzstan	*Stop COVID-19 kg*
Latvia	*Apturi Covid Latvia*
Malaysia	*MyTrace*
Mexico	*CovidRadar.mx, Plan Jalisco Covid-19*
New Zealand	*NZ COVID Tracer*
North Macedonia	*StopKorona!*
Norway	*Smittestopp*
Peru	*PerúEnTusManos – Detén el avance del COVID19*
Philippines	*WeTrace*
Poland	*Kwarantanna domowa*
Qatar	*Ehteraz*
Saudi Arabia	*Tabaud*
Singapore	*Contact Tracer, TraceTogether*
Slovakia	*Zostaň Zdravý*
South Africa	*Covi-ID*
South Korea	*Corona 100 m*
Spain	*COVID-19.eus*
Switzerland	*SwissCovid*
Thailand	*MorChana*
Tunisia	*E7mi*
United States of America	*Contact Tracer, SafePaths, HEALTHLYNKED COVID-19 Tracker, Healthy Together—COVID-19*
United Arab Emirates	*TraceCovid*
Ukraine	*Action at Home*
United Kingdom	*NHS Covid-19*
Uruguay	*Coronavirus UY*
Vietnam	*Bluezone—Electronic mask*

## Footnotes

1 Sars-CoV-2 is a mutation of a corona virus known to cause severe disease in the human body; see e.g. Corman et al. (2018), Andersen et al. (2020). Note, however, that there exists disunity among scientists with regards the severity of Sars-CoV-2.

2 By the end of April, 190 countries and territories had closed educational institutions. As of 1 August 2020, 106 countries still observe a nation-wide school closure (UNESCO 2020).

3 An example is Switzerland, see e.g. Schweizerische Eidgenossenschaft BAG (2020a).

4 E.g. in Austria, during the lockdown, the total number of unemployed people rose up to 500.000 (12.2%) within one month, reaching its highest level since 1946 (AMS 2020). The EU unemployment rate rose up to 7.2%, compared to 7.1% in June, and to a 12-year-low of 6.8% in March 2020 (Eurostat 2020).

5 Note, e.g., that, in Germany, the treatment of heart attacks sank by up to 40% (Garcia et al. 2020; Slagman et al. 2020).

6 E.g. New Zealand, Spain, Germany, South Korea, India, Iran, Hungary, Singapore, Dubai, Panama, Peru, Thailand (Kaplan et al. 2020).

7 Belgium, e.g., is re-imposing drastic social distancing measures in order to avoid a new general lockdown. For Belgium citizens, contacts outside family circles must be limited to the same five people over the month of August 2020. See e.g. Van Dorpe and Furlong (2020).

8 E.g. California (Somerville 2020) and Victoria in Australia (Picheta 2020).

9 Art. 13 (1) Universal Declaration of Human Rights (UNDHR), art. 12 (2) International Covenant on Civil and Political Rights (ICCPR).

10 Art. 20 (1) UDHR, art. 21 ICCPR.

11 Art. 26 (1) UDHR.

12 Art. 25 (1) UDHR.

13 Art. 23 (1) UDHR.

14 E.g., between March and May 2020, widespread layoffs and furloughs have prompted about 20% of the US labor force to file for unemployment benefits. See e.g. Tappe (2020).

15 However, the findings are not yet peer-reviewed published (Kunkel 2020).

16 Covid-19 has brought up war rhetoric, see e.g. Goninet (2020).

17 Art. 12 UDHR, art. 17 ICCPR.

18 This section partly relies on research conducted by Woodhams (2020).

19 A list of contact tracing apps per country can be found in the “Appendix”.

20 See e.g. the tracking-app developed with the support of Swiss researches at the EPFL in Lausanne, Handelszeitung (2020).

21 See tweet by Dr. Vesselin Bontchev from 24 March 2020: https://twitter.com/VessOnSecurity/status/1242503942409519106?s=20. Accessed 2 April 2020.

22 The Jordanian app ‘Cradar’ is designed to allow citizens to inform Jordanian authorities about unauthorized gatherings.

23 See an announcement by the Russian Government ordering the Ministry of Communications to develop a new contact tracing system to help monitor citizens thought to have come into contact with those that have the virus (Russian Government 2020).

24 See the tweet from Spectrum News NY1: https://twitter.com/NY1/status/1243502731408670720. Accessed 2 April 2020.

25 See the tweet from the Derbyshire police: https://twitter.com/DerbysPolice/status/1243168931503882241. Accessed 2 April 2020.

26 See the tweet from Raphael-Antonis Stylianou, the EU Commission’s Online Communications Officer: https://twitter.com/Stylianou_EU/status/1241405641266249728?s=20. Accessed 2 April 2020.

27 See the tweet by BBC World News: https://twitter.com/BBCWorld/status/1239267152546467843?s=20. Accessed 2 April 2020.

28 India’s CG Covid-19 ePass requires citizens to register for an electronic pass to authorize travel. Users have to provide a photograph and an ID proof (Aadhar number).

29 See the tweet by Kenneth Roth from 19 March 2020: https://twitter.com/KenRoth/status/1240671686258802692?s=20. Accessed 2 April 2020.

30 Since 2019, the Bangladeshi government has shut down internet connections in its Rohingya refugee camps (HRW(2019)).

31 Art. 4 (1) ICCPR.

32 While many states have enacted what have been described as emergency laws in response to the pandemic, not all of these countries have actually declared a ‘state of emergency’ under law. Hence, governmental behavior is not uniform. E.g., Armenia, Estonia, Georgia, Latvia, Moldova and Romania have declared a state of emergency according to art. 15 of the European Convention on Human Rights (ECHR). Other countries in Europe, e.g. Italy and Spain have declared states of emergency in accordance with their constitutional provisions AFP (2020b); see also Armstrong (2020). Others, like the UK, have introduced what politicians have described as ‘emergency powers.’ The UK government, e.g., convinced parliament to pass lengthy legislation allowing extra powers in less than a week, see the Coronavirus Act 2020 of 25 March 2020 (UK Parliament 2020).

33 The restriction must be contained in a national law of general application. This law must be in force at the time the limitation is applied. The law must not be arbitrary, nor unreasonable. Further, it must be clear and accessible to the public.

34 The limitation must be somewhat ‘appropriate’ to achieve its protective function. Further, it must be the least intrusive instrument amongst all those that might achieve the desired result.

35 Peter Micek, Acces Now, Technology and human rights in times of crisis, WebDebate, DiploFoundation and Geneva Internet Platform, March 26 2020; OHCHR.

36 The tech giants Amazon, Alphabet, Apple and Facebook are already recording quarterly profit (Lopatto 2020).

37 In addressing the pandemic, ‘saving humanity’ is one of the most prominent rhetorical references of the planet’s most powerful leaders, see e.g. the Indian Prime Minister Narendra Modi (Economictimes 2020).

38 The ‘Infection fatality rate’ also considers less severe, asymptomatic courses of the disease, whereas the ‘lethality rate’ was based on the simple division of the number of fatalities by the number of proven illnesses (EBM 2020).

39 However, it must be noted that those studies are preprints and did not undergo a formal peer-review process yet.

40 This testing scheme is sometimes also called ‘mass testing’. For an overview of mass testing strategies in the EU / EEA and the UK, see, e.g., ECDC (2020). E.g., Germany tested almost 900.000 in calender week 33 (875.524), Austria 63.000 and Switzerland 73.000. The rate of individuals found positive lies at 1% in Germany (RKI 2020), 2% in Austria (BM Österreich), and 3% in Switzerland (Schweizerische Eidgenossenschaft BAG 2020b).

41 E.g. in Germany (EBM 2020).

42 The PCR-test is a molecular diagnostic test that detects the Corona virus’ genetic material, see e.g. FDA (2020).

43 E.g. in Germany and Switzerland (EBM 2020).
